# The NRG1 exon 11 missense variant is not associated with autism in the Central Valley of Costa Rica

**DOI:** 10.1186/1471-244X-7-21

**Published:** 2007-05-22

**Authors:** Lynne A McInnes, Leonid Ouchanov, Alisa Nakamine, Patricia Jimenez, Marcela Esquivel, Marietha Fallas, Silvia Monge, Pamela Bondy, Elina R Manghi

**Affiliations:** 1 Department of Psychiatry, Mount Sinai School of Medicine, New York, New York, USA; 2 Department of Human Genetics, Mount Sinai School of Medicine, New York, New York, USA; 3 Hospital Nacional de Ninos "Dr Sáenz Herrera"; CCSS, Child Developmental and Behavioral Unit, San José, Costa Rica; 4 University of Illinois at Chicago, Chicago, Illinois, USA

## Abstract

**Background:**

We are conducting a genetic study of autism in the isolated population of the Central Valley of Costa Rica (CVCR). A novel Neuregulin 1 (NRG1) missense variant (exon 11 G>T) was recently associated with psychosis and schizophrenia (SCZ) in the same population isolate.

**Methods:**

We genotyped the NRG1 exon 11 missense variant in 146 cases with autism, or autism spectrum disorder, with CVCR ancestry, and both parents when available (N = 267 parents) from 143 independent families. Additional microsatellites were genotyped to examine haplotypes bearing the exon 11 variant.

**Results:**

The NRG1 exon 11 G>T variant was found in 4/146 cases including one de novo occurrence. The frequency of the variant in case chromosomes was 0.014 and 0.045 in the parental non-transmitted chromosomes. At least 6 haplotypes extending 0.229 Mb were associated with the T allele. Three independent individuals, with no personal or family history of psychiatric disorder, shared at least a 1 megabase haplotype 5' to the T allele.

**Conclusion:**

The NRG1 exon 11 missense variant is not associated with autism in the CVCR.

## Background

We are conducting a population genetic study of autism in the CVCR [1]. Recently, Walss-Bass et al. [2] reported that they had identified a novel missense variant in the transmembrane domain of Neuregulin 1 (Val>Leu in exon 11) associated with the phenotypes of psychosis (SCZ and psychotic mood disorder) and SCZ in the CVCR. The cases apparently descended from a common ancestor who migrated to Costa Rica from Spain and was born in 1670. Genome-wide linkage studies of autism have not highlighted chromosome 8p12, where NRG1 is located, and autism is not characterized by psychosis. However, both autism and SCZ have been linked to abnormal expression of the Reelin (RELN) gene (reviewed in Fatemi 2005 [3]). Additionally, deletions of 22q11.2 are associated both with autism and psychosis [4,5] and another group demonstrated that offspring of parents with psychosis or affective disorders had a greater risk of developing autism [6]. Finally, SCZ is widely present in the relatives of our autism probands and these probands have been ascertained from the CVCR as in the Walss-Bass study. Given these data suggesting a common genetic basis for at least a subset of autism and SCZ subjects, and the shared ancestry of the autism and SCZ probands in both studies, we decided to screen our cases for the presence of the NRG1 exon 11 G>T missense variant.

## Methods

### Subjects

We are collecting autism trios with the goal of performing a population genetic study of autism in the isolated founder population of the CVCR. As this population was founded by a small number of individuals in the 16^th ^century, and grew exponentially in isolation until the 1970s, disease alleles present at the founding should be widely distributed in the current population. This project was approved under the guidelines of the Ministry of Health of Costa Rica, the Ethical committee of the National Children's Hospital in San Jose (Hospital Nacional de Ninos or HNN), and the Institutional Review Board at Mount Sinai School of Medicine in accordance with the Declaration of Helsinki. These approvals remain active.

Details of the case ascertainment and evaluation process have been previously described [1]. Briefly, families of individuals with possible autism contact us, or are contacted by us after expressing interest in the study, and are formally asked to participate using established informed consent criteria. All interviews and exams take place in the Neurodevelopmental Unit of the HNN. Possibly affected individuals and their parents are interviewed by experienced neuropediatricians using the Autism Diagnostic Interview Revised (ADI-R) [7]. The Autism Diagnostic Observation Schedule (ADOS) [8] is also administered to subjects and both of these assessments are videotaped for independent scoring by the best estimators. IQ tests are administered that are appropriate for the age and level of verbal communication of the subjects. The Vineland Adaptive Behavioral Scales [9] are administered and compared with the results of IQ tests to confirm diagnoses of mental retardation.

All subjects are evaluated with a complete medical and neurological examination, including a dermatological examination with Wood's Lamp to look for signs of tuberous sclerosis and Hypomelanosis of Ito. All subjects in this study have been reported negative for *FRAXA *mutations and subjects with mental retardation were reported to have normal G-banded karyotypes at 550-band resolution.

Subjects were included in this study if they met DSM IV-TR [10] criteria for autism in all three symptom domains (social interaction, communication or language, and behavioural abnormalities) as measured by the ADI-R and the ADOS. Additionally, age of onset for at least one symptom domain must have been less than 36 months. A diagnosis of autism spectrum was given to individuals who met ADI-R criteria but had an age of onset greater than 36 months, or were no more than one point below ADI-R criteria for autism in the social domain, and in either the communication or repetitive behaviour domains, but not in both, or if they met full criteria using the ADI-R but were one point below the cut-off score for autism in one domain of the ADOS. We also included subjects with a DSM IV-TR diagnosis of Asperger's Syndrome. Although we will not include probands with a DSM IV-TR diagnosis of Pervasive Developmental Disorder Not Otherwise Specified (PDD-NOS) in a future whole genome association study of autism, we did include them in this exploratory study. Subjects with a nonverbal IQ <35 were excluded unless they had an adaptive behaviour score much higher than the IQ measure. This is because there are no local Costa Rican norms for IQ testing and thus IQ may be underestimated in this population [1].

All individuals also had to have CVCR ancestry defined as having at least 6/8 great-grandparents (GGPs) born in the CVCR, or all 4 GGPs from either the maternal or paternal side (in which case only transmission from the parent with CVCR ancestry is considered). Furthermore, individuals cannot be related by fewer than 6 generations, although we did genotype siblings, if affected, in the current study. The sample genotyped for this study includes 143 independent families. There were 129 cases with autism, 67% of which also had a diagnosis of mental retardation. The rest of the cases did not have mental retardation and included N = 7 with autism spectrum disorder, N = 4 with PDD-NOS, and N = 3 with Asperger's Syndrome. Three autism cases also had siblings with either PDD-NOS (2), or autism (1). The mean age of the subjects was 5.75 years and the range was 3–13. Sixteen of the 143 cases were female. The three multiplex families consisted of two male sib pairs and one male:female sib pair.

### Genotyping

A TaqMan SNP genotyping assay was designed using ABI Assay-by-Design File Builder Version 2.0 from the following genomic sequence:

GAACATGGACAATGTCATGCAGCATGCCCACTGTTTGGTTGTAGTCAGTCCTGGCAAGTGGAAGTGACCTGTGATGACATCTGCTCTCATCCCTTTCCAGAGGCGGAGGAGCTGTACCAGAAGAGAGTGCTGACCATAACCGGCATCTGCATCGCCCTCCTTGTGGTCGGCATCATGTGT [**G/T**]TGGTGGCCTACTGCAAAACCAAGTAAACCTTCTTTCTCCATGCCTTTCTCTCTCCTTCATGCAGAGACAGCTTAGATGGCCAGGGCTTTGCAGAATCTGAGCTCCACAGCCTAGTCTTGGGG.

The sequences used for the amplification primers are as follows: Forward primer, TGCATCGCCCTCCTTGTG; Reverse primer, AGAAAGGCATGGAGAAAGAAGGTTT.

Identification of the G allele was accomplished using the probe CACCACACACATGAT-8 labelled with VIC. Identification of the T allele was performed using CCACCAAACACATGAT-8 labelled with FAM. The SNP Genotyping Assay Mix was obtained from Applied Biosystems (Foster City, CA) at a concentration of 40×, and diluted to 20× with 1× TE. For each reaction, 2.50 μl of 2× TaqMan Universal PCR Master Mix, No AmpErase UNG (Applied Biosystems, Foster City, CA), 0.25 ul of 20× SNP Genotyping Assay Mix, and 2.25 μl of genomic DNA at 10 ng/μl were used for a total reaction volume of 5 μl using 384-well plates. The plates were run in the ABI 7900 thermal cycler with the following conditions: 95°C for 10 minutes hold, then 40 cycles of 15 second denaturation at 92°C and 1 minute of annealing/extension at 60°C. At the completion of the amplification and the absolute quantification read, the allelic discrimination assay read was performed to generate the allelic discrimination read file to be analyzed on SDS 2.1.

Microsatellite markers for haplotype analysis included 317J8-2123, D8S1810, 420M9-1395 and 487-2. Details regarding genotyping of these microsatellites are available upon request. Note that the order of these markers has changed in the Human Genome Browser March 2006 assembly since the publication of NRG1 haplotypes by Li et al. [10]. D8S1810 and 420M9-1395 have switched positions. Details regarding the microsatellite markers we utilized are available at deCODE Genetics [14].

## Results

### Frequency of the exon 11 missense variant in cases and parental non-transmitted chromosomes

**Figure 1:**
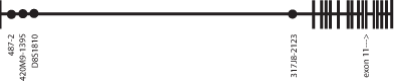
**Position of the microsatellite markers genotyped in NRG1**. Diagram not to scale with regards to the position of exons.

The exon 11 missense variant was present in two cases diagnosed with autism, one case diagnosed with an autism spectrum disorder (one point below one domain of the ADI-R), and one case diagnosed with PDD NOS, in addition to co-morbid attention deficit hyperactivity disorder and a tongue tic (Case 129). The allele was transmitted from the mother in all cases but appears to have occurred de novo in Case 129. The missense allele was also present in 12 unrelated, other than by marriage, parental individuals, 7 fathers and 5 mothers, from 11 families who did not transmit the allele to cases. To be clear, both parents of Case 25 carry the T allele and they are not related to each other by fewer than 6 generations. The frequency of the allele in the non-transmitted chromosomes was therefore 12/267 = 0.045, remarkably close to the frequency estimate of 0.042 by Walss-Bass et al [2]. Haplotype analysis revealed the presence of 6 haplotypes carrying the missense T allele when considering only the closest microsatellite 317J8-2123, 0.229 Mb proximal to the exon 11 missense variant (Table 1). Please see Figure 1 for the position of microsatellite markers typed in relation to the exon 11 variant. After microsatellite 317J8-2123, the next closest markers are clustered together with D8S1810 at ~0.964 Mb upstream followed by 420M9-1395 and then 487-2 ~1.04 Mb upstream from the first marker. These three 5' markers were typed as most associations to NRG1 with SCZ have been to markers in this region. Three parental chromosomes from 3 independent families (one transmitted to a case and the other two non-transmitted) shared alleles at all 4 microsatellite markers typed over a distance of ~1.04 Mb (Table 1). Two other independent parental non-transmitted chromosomes also may share a different ~1.04 Mb haplotype however phase is uncertain.

**Table 1:** Microsatellite haplotypes observed in exon 11 missense variant carriers.

Family ID	A/B/C/D	487-2	D8S1810	420M9-1395	317J8-2123	Exon 11 G>T	T/NT	Hap#	Comment
AU025	25B	247.5	194	190	240	T	NT	1	
AU025	25C	235.7	192	202	247	T	NT	2	Sister with bipolar disorder
AU027	27C	*247.5*	*194*	*(190)*	*246*	*T*	*NT*	*3*	Possible extended hap
AU044	44C	*247.5*	*(194)*	*190*	*246*	*T*	*NT*	*3*	Possible extended hap
AU058	**58A**	245.7	176	181	247	T	T	2	
AU058	58C	245.7	176	181	247	T	T	2	
AU058	58D2	245.7	176	181	247	T	T	2	
AU059	**59A**	235.7	196	202	242	T	T	4	
AU059	59C	235.7	196	202	242	T	T	4	
AU061	61B	***237.7***	***178***	***181***	***242***	***T***	NT	4	Extended hap
AU071	71B	***237.7***	***178***	***181***	***242***	***T***	NT	4	Extended hap
AU090	**90A**	***237.7***	***178***	***181***	***242***	***T***	T	4	Extended hap
AU090	90C	***237.7***	***178***	***181***	***242***	***T***	T	4	
AU089	89C	246.4	176	***181***	***242***	***T***	NT	4	Extended hap w recomb?
AU113	113C	253.1	178	186	242	T	NT	4	Brother with SCZ
AU129	**129A**	235.7	194	202	246	T	NT	3	De novo in case
AU143	143B	243.8	194	202	253	T	NT	5	
AU164	164B	241.8	178	186	253	T	NT	5	
AU165	165B	239.7	196	190	238	T	NT	6	

The first column contains the family ID number of the autism proband. The second column indicates the family member status, for example 25A signifies the case, 25B signifies the father, 25C signifies the mother and 58D2 indicates the second sibling of case 58. The next 4 columns contain alleles from the 4 microsatellites genotyped to determine which haplotypes are associated with the rare 'T' allele. Alleles were binned where possible with the exception of marker 478–642 wherein the allele is called as reported by the software. Brackets around an allele mean that phase could not be determined with certainty. The "T/NT" column indicates whether the haplotype was transmitted, or not transmitted, from a parent to a case, or the sibling of a case. The "Hap#" column indicates the number of different haplotype backgrounds associated with the 'T' allele considering only the closest microsatellite 317J8-2123 (0.229 Mb away). The comment column notes extended haplotypes > ~1 Mb A question mark next to the haplotype number indicates that the haplotype background could not be determined with certainty.

### Psychiatric history of exon 11 missense variant carriers

We take a careful family psychiatric history on first, second and third degree relatives of all probands. Therefore, we reviewed the psychiatric history of all cases as well as persons carrying the T allele to look for the presence of psychotic or affective disorders. None of the 12 'T' carrier non-transmitting parents had a psychiatric disorder. However, AU025C, a 40 year old mother did have a sister with manic-depression and, AU113C, a 38 year old mother, did have a brother who had been hospitalized with SCZ (unfortunately DNA samples could not be obtained on these subjects). This inheritance pattern could represent incomplete penetrance in carriers of the missense allele. However, 113C and 25C did not share a haplotype with each other. Of note, 7 trios not segregating the T allele had a blood-related aunt or uncle with schizophrenia, which were not available for testing. An 8^th ^case had a parent with SCZ who did not carry the T allele and a parental sib with SCZ whose carrier status is unknown. A 9^th ^autism case had more than one first degree relative with SCZ, neither of whom carried the T allele. None of the three T-allele transmitting parents had a personal or family history of psychotic or affective disorders. Finally, Case 129 appears to carry a de novo occurrence of the T allele as we have observed no other non-mendelian transmissions from either parent to Case 129 in a panel of markers spanning chromosomes 15, 7, 17, 21 and 4 which should effectively rule out non-paternity.

## Discussion

Our major conclusion is that the NRG1 missense variant is not associated with autism in our sample. However, can we conclude anything about a relationship of this allele to SCZ or psychosis from our results?

Walss-Bass et al. [12] first genotyped 6 of 7 markers previously observed to be associated with SCZ in Iceland [13] in a sample of 134 trios with a history of psychosis including 94 cases diagnosed with SCZ, 16 cases with schizoaffective bipolar disorder, 4 cases with schizoaffective disorder depressed, 5 cases with major depressive episode accompanied by psychosis and 3 cases with psychosis not otherwise specified. They further stratified their sample into SCZ (N = 114), manic psychosis (N = 63), and non-manic psychosis cases. They did not find statistically significant evidence for over-transmission of alleles or haplotypes containing markers from the 'deCode' haplotype after correcting for multiple testing. However, they reported a trend (p < 0.05 uncorrected for multiple testing) towards over transmission of the most common 4 marker haplotype that they observed in their sample (f = 0.168), to cases with manic psychosis. Similarly, alleles at the two microsatellite markers in this core haplotype were not significantly associated with manic psychosis after correction for multiple testing.

Walss-Bass et al. [2] subsequently selected the 12 individuals with a history of SCZ or psychosis and mania that carried alleles representing the most common haplotype observed in cases for sequencing. They found a G>T polymorphism in exon 11 resulting in a Val>Leu missense change observed in 1 of the 12 cases. However, this exon 11 variant was not in LD with the haplotype that they used to guide their search for a disease polymorphism so it may represent a serendipidous finding. They next genotyped the exon 11 variant in 378 individuals from 151 different CVCR families, 142 of whom were affected with psychosis. The T allele was transmitted 13 times from heterozygous parents to affected individuals and not transmitted 4 times yielding a significant association with psychosis (Z = 2.810, p = 0.0049) and with SCZ (Z = 2.383, p = 0.0191) using the Family-based Association Test (FBAT). The authors then found genealogical connections linking the families transmitting the T-allele to one common Spanish founder. Our data do show evidence of a possible founder effect surrounding the T-allele in 3 independent individuals (and possibly in another 2 independent individuals) however none of those families were segregating SCZ.

## Conclusion

In summary, our data does not support a relationship of the NRG1 exon 11 missense variant with either autism or SCZ in the CVCR, although it does not reduce support for the previous association with SCZ either. Further studies examining the frequency of the exon 11 T allele in control individuals from the CVCR, and LD in the vicinity of the exon 11 variant should help determine whether the NRG1 allele actually does contribute to psychosis in this population.

## Abbreviations

CVCR Central Valley of Costa Rica, NRG1 Neuregulin 1, schizophrenia SCZ.

## Competing interests

The author(s) declare that they have no competing interests.

## Authors' contributions

LAM analyzed the genotyping data and wrote the manuscript, LO and AN performed the microsatellite and Taqman genotyping and assisted with analysis of the genotyping data. PJG, ERM, ME, SMM and MFD performed the clinical evaluation of the probands and reviewed the case histories of all 'T-allele' carriers. All authors read and approved of the manuscript.

## Pre-publication history

The pre-publication history for this paper can be accessed here:


